# Comparison of Pretreatment in European Society of Cardiology Acute Coronary Syndrome Guidelines

**DOI:** 10.5811/westjem.43528

**Published:** 2025-10-22

**Authors:** İsmail Ataş, Mümin Murat Yazıcı, Ahmet Nurhak Çakır, Nurullah Parça, Utku Sap Cerit, Meryem Kaçan, Özlem Bilir

**Affiliations:** * Recep Tayyip Erdoğan University Training and Research Hospital, Department of Emergency Medicine, Rize, Türkiye; † Rize State Hospital, Department of Emergency Medicine, Rize, Türkiye

## Abstract

**Introduction:**

Most patients with acute coronary syndrome (ACS) die before hospitalization. Early diagnosis and effective interventions can prevent the disease from worsening. In this single-center, retrospective study we aimed to investigate the appropriateness of the pretreatment of patients referred to the emergency department of our hospital, a percutaneous cardiac intervention (PCI) center, with a prediagnosis of ACS under the previously published European Society of Cardiology guidelines (2017 and 2020) and the new guidelines published in 2023.

**Methods:**

Based on the date of publication of the European Society of Cardiology’s most recent ACS guidelines (August 25, 2023), we divided patients admitted between August 25, 2022–August 24, 2024, into two groups: patients who were evaluated and received pretreatment under the previous guidelines; and patients who were evaluated and received pretreatment under the new guidelines.

**Results:**

Of 1,675 patients screened for enrollment who were referred to our PCI center with prediagnosis of ACS, after exclusion criteria, we report on 1,450 (86.6%). Pretreatment (before PCI) compliance rate with all aspects of the previous and new guidelines was low, at 9.8%. Study patients were 69.9% (n = 1,013) male with mean age of 63.9 ± 13.0 years. Comparing the compliance rate between the new versus previous guidelines, for individual components, we found better compliance for aspirin administration (72.6 vs. 66.2%) and anticoagulants (40.3 vs. 22.7%), while for P2Y12 inhibitors, we found lower compliance (58.9 vs. 70.0%, all p< .001). For the subset of patients with ST-elevation myocardial infarction, P2Y12 inhibitors were used less appropriately under the new vs. previous guidelines (31.4 vs. 55.0%, p < .001).

**Conclusion:**

The compliance rates with the previous and new guidelines for ACS pretreatment by physicians working in hospitals without PCI centers were low. Pretreatment compliance during the new guideline period was lower than compliance during the prior guideline period.

## INTRODUCTION

Acute coronary syndrome (ACS) is a clinical condition that causes myocardial ischemia and infarction due to partial or complete occlusion of the coronary vessels by atherosclerotic plaque. The diagnosis is based on clinical findings, electrocardiographic (ECG) evaluation, and cardiac enzyme results. Despite advances in the diagnosis and treatment of ACS, the mortality rate from cardiovascular disease is very high worldwide. Half of these deaths are due to ischemic heart disease.^1^,^2^ Most patients with ACS die before hospitalization. The cause of mortality is fatal dysrhythmias triggered by ischemia. Early diagnosis of ACS and effective interventions can prevent the disease from worsening.

Patients diagnosed with ACS are placed into three categories according to clinical, ECG, and cardiac enzyme results: ST-elevation myocardial infarction (STEMI); non-ST elevation acute coronary syndrome (NSTEMI); and unstable angina pectoris (USAP). In recent years, STEMI rates have been decreasing, and NSTEMI rates have been increasing. This is because of the widespread use of high-sensitivity troponin.^3^

The main aim of treatment is to ensure the patency of the coronary vessels and myocardial perfusion at an early stage and to prevent myocardial damage and related complications.^4^–^6^ For this purpose, patients diagnosed with ACS should receive primary percutaneous intervention (PCI) within 60–90 minutes.^7^ The sooner perfusion is achieved after the onset of the thrombotic process, the easier it is to achieve the treatment goal. Pretreatment until reaching PCI is also of great importance in the management and treatment of ACS. For this purpose, antithrombotic and anticoagulant therapies are recommended for diagnosed patients.^8^–^10^ The European Society of Cardiology (ESC) periodically publishes guidelines for the management of ACS that are recognized and followed by all countries. Increased guideline adherence in treating ACS in hospitals potentially reduces major adverse cardiac events, heart failure, and mortality. The updated ESC guideline released on August 25, 2023, included some changes regarding the pretreatment of patients diagnosed with ACS.^11^

In this single-center, retrospective study we aimed to investigate the appropriateness of the pretreatment of patients referred to the emergency department (ED) of our hospital, a PCI center, with a prediagnosis of ACS under the previous and new ESC guidelines.

## METHODS

### Study Design and Setting

We conducted an analysis of patients referred to the ED of a tertiary-care hospital, the only PCI center in the province, with ACS prediagnosis from external centers between August 25, 2022–August 24, 2024. Approval for the study was obtained from the local ethics committee on January 22, 2025 (decision no. 2025/15).

### Patient Selection and Data Collection

**Figure 1:**
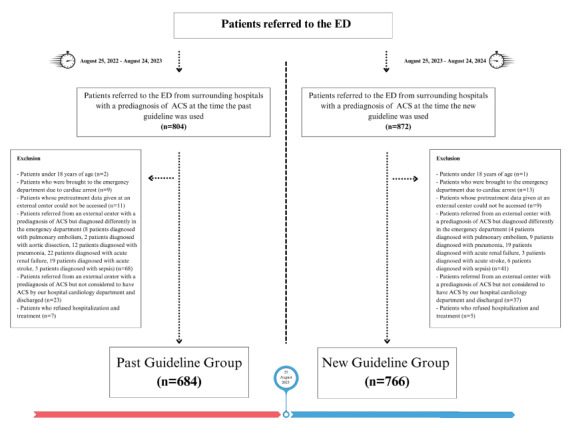
Patient flow chart grouped according to the previous and new European Society of Cardiology guidelines for treating acute coronary syndrome, showing inclusion and exclusion criteria. *ACS*, acute coronary syndrome; *ED*, emergency department.

The patient group included patients who were referred to our ED from external centers with a prediagnosis of ACS and were considered to have ACS by the cardiologists at our hospital, and admitted to the cardiology service or coronary intensive care unit (ICU). We excluded patients based on the exclusion criteria described in Figure 1. We divided patients admitted between August 25, 2022,–August 24, 2023 into two groups based on the date of the ESC ACS guideline published on August 25, 2023: the first group included patients who were evaluated and received pretreatment under the previous guidelines; and the second group included patients who were evaluated and received pretreatment under the new guidelines. We included in the study 684 patients evaluated while the previous guidelines were in effect and 766 patients evaluated in accord with the most recent guidelines after the exclusion criteria were applied.

**Population Health Research Capsule**

What do we already know about this issue?

*Pretreatment adherence to acute coronary syndrome (ACS) guidelines improves early outcomes, yet compliance is often suboptimal in non-percutaneous cardiac intervention (PCI) centers.*

What was the research question?

*Has pretreatment compliance changed under the 2023 ACS guideline in patients referred from non-PCI centers?*

What was the major finding of the study?

*Compliance dropped from 11.5% to 8.2% under the new guidelines (P = .03), and conformity to P2Y12 inhibitors in STEMI declined (55.0 to 31.4%, P = .001).*

How does this improve population health?

*Identifying gaps in guideline adherence highlights the need for ongoing training to optimize ACS pretreatment and outcomes.*

### Study Protocol

We studied patients with a prediagnosis of ACS who were evaluated by general practitioners or emergency physicianss in external centers and referred to the ED of a tertiary-care hospital, the only PCI center in the province. Patients admitted to the ED were initially evaluated by emergency medicine (EM) residents or attending emergency physicians. The remaining patients were seen by cardiologists. We excluded patients who were not considered to have ACS and were discharged. The remaining patients were admitted to the cardiology service or cardiology ICU with diagnoses of STEMI, NSTEMI, or USAP. These hospitalized patients constituted the participants in the study.

We collected patient data using the computer-based hospital information management system standard for all provincial hospitals, along with patient files and referral reports. We recorded demographic information (age, sex, and comorbidities), final diagnosis, pretreatments given at an external center (aspirin, P2Y12 inhibitors, anticoagulants), reperfusion treatments (medical, stent, coronary artery bypass), and mortality (at 48 hours and 30 days).

Three EM residents and one attending emergency physician scanned all data for the referred patients for two years in six-month periods and created data tables. The first year was considered the period when the prior ESC guidelines were in effect, and data were collected by two EM residents. The last two six-month periods were considered to be the time frame when the new guidelines were in effect, and data were collected by one EM resident and one attending emergency physician. All four physicians who collected the data were blinded to each other. The collected data were evaluated and statistically analyzed by two different emergency care specialists who were blinded to each other and to the other physicians who collected the data. To ensure optimization for retrospective review in EM research papers, we applied the following method criteria: “abstractor training; case selection criteria; variable definition; abstraction forms; performance monitoring; blind to hypothesis; medical record identification; sampling method; data management plan for missing data; institutional review board approval.”^12^ In addition, since this study was retrospective observational, we applied the Strengthening the Reporting of Observational Studies in Epidemiology guidelines to ensure optimization.^13^

### Study Objective

The primary objective of the study was to investigate the compliance of physicians working in hospitals without PCI centers under the previous and new ESC guidelines for the application of ACS pretreatment.

### Statistical Analysis

We performed all analyses using Jamovi v1.6 statistical software (The Jamovi Project [2021] Computer Software, Sydney, Australia). Categorical data are expressed as frequencies (n) and percentages. Normally distributed continuous variables are presented as mean plus standard deviation, and non-normally distributed data are presented as median and interquartile range (IQR). The normality of distribution was assessed using the Shapiro-Wilk test. We compared continuous variables in independent groups using the *t*-test in the case of normal distribution and the Mann-Whitney U test in the case of non-normal distribution. Comparisons of categorical data were conducted using the chi-squared test. In all statistical analyses, *P* values < .05 were considered significant.

## RESULTS

The study population consisted of 1,450 patients (after exclusion criteria were applied to 1,676 patients) referred to our PCI center with a prediagnosis of ACS. We included 684 from the period during which the previous guidelines were in effect, and 766 patients from the period during which the new guidelines were in effect. Of the patients included in the study, 1,013 (69.9%) were male. The mean age of the patients was 63.9 ± 13.0 years. The most common comorbidities were hypertension (75.5%), coronary artery disease (38.6%), and diabetes mellitus (34.1%). In patients hospitalized with a prediagnosis of ACS, the most common diagnosis was NSTEMI (55.4%). The rates of STEMI and USAP diagnoses were 30% and 14.6%, respectively. Stents were implanted in 47.9% of inpatients. Aspirin (69.6%) was the medication most commonly given to patients for the pretreatment of ACS in external centers; P2Y12 inhibitors were given to 64.1% of the patients, and anticoagulants were given to 32%. The mortality rate was 2.6% within 48 hours and 4.8% within 30 days. The pretreatment compliance rate with the previous and new guidelines was 9.8%. Demographic data for the patients and their baseline characteristics are shown in Table 1.

**Figure 2:**
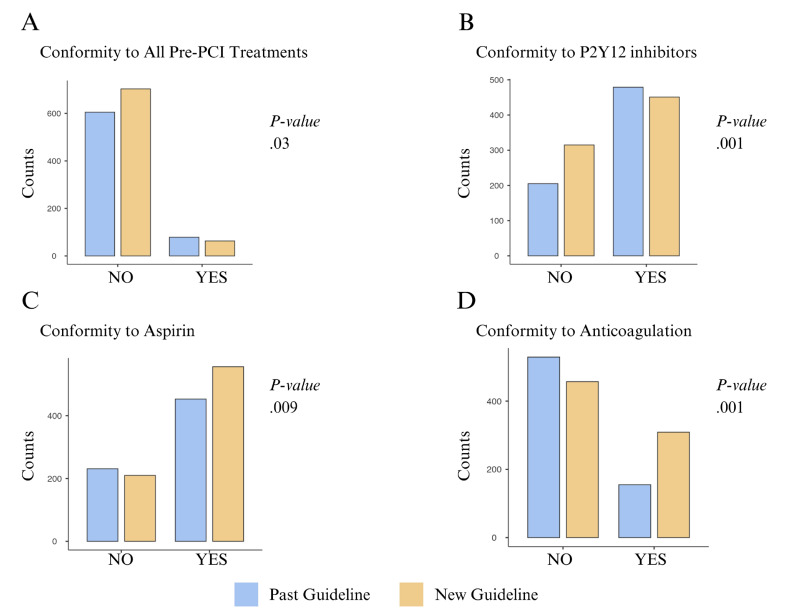
Bar plot of the conformity of the percutaneous cardiac intervention treatments with the between in groups (previous and new European Society of Cardiology guidelines). *PCI*, percutaneous cardiac intervention.

An evaluation of the pretreatment for all patients showed that the compliance rate for the administration of aspirin and anticoagulants under the new guidelines was significantly higher than the compliance rate under the previous guidelines (*P* < .001, *P* < .001, respectively). while the compliance rate for the administration of P2Y12 inhibitors was significantly lower under the new guidelines compared with the compliance rate under the previous guidelines (*P* = .001). An analysis of the compliance of all pretreatments with the guidelines showed that the rate of compliance with the new guidelines was lower than the rate of compliance with the previous guidelines (*P* = .03) (Figure 2).

No statistical significance was found between the pretreatment given to USAP patients under the previous guidelines and that given to USAP patients under the new guidelines in terms of aspirin, P2Y12 inhibitor, and anticoagulant use. Likewise, no statistically significant difference was found between the two periods in terms of compliance rates with pretreatment guidelines (*P* = .67). When we compared compliance with the previous guidelines for pretreatments given to patients with NSTEMI with compliance under the new guidelines, the compliance rate of anticoagulation with the new guidelines was statistically higher than the compliance rate with the previous guidelines (*P* = .001), whereas no statistical difference was found between the two periods in the use of aspirin and P2Y12 (*P* = .71 and *P* = .26, respectively). No statistically significant difference was found when all pretreatments were compared according to the guidelines (*P* = .24).

When the compliance rate of pretreatments given to patients with STEMI under the two different guidelines was compared separately, we found that aspirin and anticoagulants were used more appropriately under the new guidelines (*P* = .001 and *P* = .001, respectively), while P2Y12 inhibitors were used less appropriately (*P* = .001) under the new guidelines. The compliance rate of all pretreatments with the new guidelines was statistically lower than the compliance rate with the previous guidelines (*P* = .001). Details of the compliance of pretreatments given to ACS patients with the previous and new guidelines are shown in Table 2.

## DISCUSSION

Pretreatment is an important component in the management of patients presenting with ACS. The treatment plan varies according to the patient’s comorbidities, bleeding status, diagnostic group (STEMI, NSTEMI, or USAP), and time to PCI. An aspirin-loading dose should be started as soon as possible in all ACS patients, regardless of the diagnostic group.^14^ Parenteral anticoagulation is recommended for all patients with ACS at the time of diagnosis.^15^,^16^ Under both the previous and new guidelines, routine pretreatment with P2Y12 inhibitors is not recommended for patients with NST-ACS (NSTEMI and USAP) with unknown coronary anatomy and planned early invasive management (< 24 hours).^8^,^17^,^18^ The August 25, 2023, update to the ESC ACS guidelines contained some pretreatment changes. In the previous guideline, “Routine pretreatment with P2Y12 inhibitors should be performed at the time of diagnosis in STEMI” was recommended class 1, evidence level A, whereas in the new guideline, the recommendation is class IIb, evidence level B.^4^,^11^,^19^ As a result of this update, clinicians have commented that pretreatment with P2Y12 inhibitors in STEMI patients should be left to the cardiologists at the PCI center rather than given at the time of diagnosis. The impact of this change is also evident in our study. There was no significant difference between the pretreatment of patients under the previous and new guidelines in terms of compliance with the guidelines for the use of P2Y12 inhibitors in patients with USAP and NSTEMI, whereas under the new guidelines, P2Y12 inhibitors were used less appropriately for STEMI patients.

The rate of appropriate pretreatment with a combination of aspirin, P2Y12 inhibitors, and anticoagulants was 9.8% in all patients included in the study: 11.5% in patients treated during the period when the previous guideline was in effect, and 8.2% in patients treated after the new guideline came into effect. These results suggest that clinicians working in hospitals without PCI centers have deficiencies in guideline-appropriate pretreatment. The lower compliance with pretreatments specified in the new guidelines compared with compliance under the previous guidelines indicates that adaptation to the new guideline has not yet occurred. It is known that the knowledge and performance of clinicians increase with continuous theoretical and practical training.^20^,^21^ Clinicians working in noneducational hospitals should also be made aware of the current guidelines. In a multicenter study on inhospital ACS management guidelines, PCI within 90 minutes, cardiac risk scoring tools, and secondary prevention medications were the three main factors that reduced heart failure and mortality in patients.^22^ Many studies have focused on these factors. In our study we took a different approach and looked at guideline compliance of ACS pretreatment but did not focus on patients’ clinical outcomes. In this respect, our study is unique and has the potential to be improved.

The biggest change in the new guidelines regarding pretreatment is related to the use of P2Y12 inhibitors in STEMI. In our study, we found that the appropriate use of P2Y12 inhibitors in the pretreatment of patients with STEMI decreased under the new guidelines. The change in the recommendation class and level of evidence for the use of P2Y12 inhibitors in STEMI in the new guidelines has caused confusion among clinicians. The scientific community has responded to this change with a range of opinions. Per the Dutch Society of Cardiology, based on the PLATO and TRITON studies and considering the current healthcare logistics in the Netherlands and the fact that PCI procedures are usually performed with the radial approach, it is thought that the risk of bleeding may be low and, therefore, the routine use of P2Y12 inhibitors in pretreatment can be continued.^23^–^25^ Considering that the risk of bleeding may increase in countries with poor logistic conditions (such as rugged terrain and long distances) and in countries where the femoral approach is preferred, it is better not to apply pretreatment with P2Y12 inhibitors in STEMI.

Per the ATLANTIC study, using P2Y12 inhibitors in premedication in STEMI patients had no significant effect on thrombolysis in myocardial infarction or ST-segment elevation resolution before the interventional procedure.^8^ However, it may increase the risk of minor and major bleeding. We did not investigate whether the use of P2Y12 inhibitors in the premedication of patients with STEMI caused the expected bleeding complications. However, further studies on this subject are needed. In our study, we found that although the appropriate use of P2Y12 inhibitors decreased with the implementation of the new guidelines, the appropriate use of aspirin and anticoagulants increased significantly. The fact that the recommendation class and level of evidence for aspirin and anticoagulants have not changed in the new guidelines may have caused clinicians to use these drugs with more confidence.

We found no statistical difference between the previous and new guideline periods in terms of the appropriate use of P2Y12 inhibitors in the pretreatment of patients with NST-ACS (NSTEMI and USAP). The 2014 ACCOAST study, found that P2Y12 inhibitors given to NST-ACS patients in pretreatment were not associated with a decrease in any ischemic event, including mortality, compared to the group not given P2Y12 inhibitors, and caused an increase in bleeding risk.^26^ In the ESC 2015 guidelines published following this study, the recommendation class of P2Y12 inhibitors in the pretreatment of NST-ACS was downgraded from I to III.^27^ A 10-year cohort study conducted by Ueyama et al found no statistical difference between NST-ACS patients given P2Y12 and patients not given P2Y12 in terms of mortality, major bleeding, and re-myocardial infarction, and the duration of hospitalization was prolonged in the group given P2Y12.^28^ The fact that the new guidelines are the same as the previous guidelines in this regard has ensured that clinicians continue to comply with the guidelines.

In our study, we found that aspirin (69.6%) was the most commonly used drug in pretreatment in all patients. In patients with USAP, NSTEMI, and STEMI, the rates of aspirin use were 52.1%, 67.5%, and 81.8%, respectively. This finding suggests that the practice of using aspirin increases with the increasing severity of the diagnosis. However, the two large, randomized controlled trials, ACUITY and HORIZONS-AMI, found that aspirin use in pretreatment was associated with a decrease in 30-day mortality in NST-ACS patients but not in STEMI patients.^29^–^31^ Therefore, aspirin use in pretreatment should be given more importance, especially in patients with NST-ACS. We also found that the rates of use of P2Y12 inhibitors in pretreatment in patients with USAP, NSTEMI, and STEMI were 14.2%, 29%, and 61%, respectively. The use of P2Y12 inhibitors in STEMI was significantly greater than in USAP and NSTEMI. The rates of use of P2Y12 inhibitors in STEMI during the previous guideline period compared with the new guideline period were 55% and 68.6%, respectively.

Although the level of evidence for the use of P2Y12 inhibitors in the pretreatment of STEMI decreased in the new guidelines, the rate of use increased. This is another finding indicating that clinicians have not yet adapted to the new guidelines. The restriction of the use of P2Y12 inhibitors in the pretreatment of NST-ACS, starting with the previous guidelines, may be the reason for the low utilization rates. We think that the use of P2Y12 inhibitors in STEMI will decrease over the coming years and, therefore, studies on this subject should be conducted.

In our study, the rates of anticoagulant use in pretreatment in patients with USAP, NSTEMI, and STEMI were 7.6%, 27.5%, and 52.2%, respectively. As with aspirin and P2Y12 inhibitors, we saw that clinicians were using anticoagulants in pretreatment more frequently with the increasing severity of the diagnosis. A meta-analysis conducted by Oler et al of six randomized controlled trials showed that the addition of heparin to aspirin in USAP and NSTEMI patients resulted in a 33% reduction in mortality and ischemic outcomes.^32^ The most recent guidelines recommend giving anticoagulation to all ACS patients at the time of diagnosis, and the recommendation class and level of evidence is 1A. Based on the results of the current study, clinicians should be strongly encouraged to give anticoagulation in pretreatment, especially to USAP and NSTEMI patients.

Among the reasons for the decrease in compliance with current guidelines, we can particularly mention the lack of up-to-date training on guidelines among clinicians who do not work in teaching clinics and the fact that guidelines are only published in English. To eliminate these problems, the ESC needs to train instructors who will provide accredited training on guidelines in various countries. At the same time, translations of the guidelines into the most widely used languages worldwide (eg, German, Spanish, French, Chinese) should be published simultaneously with the original language version, thereby eliminating language barriers and enabling more clinicians to access the latest guidelines.

## LIMITATIONS

Our study has several strengths, including a clear data-extraction protocol, blinding procedures, and the high number of patients included (N = 1,450). However, this study has some limitations. The first limitation is the difficulty of data collection and the limited causal inferences due to the use of a retrospective study design. To overcome this challenge and to minimize selection bias, we used objective criteria for case selection. Another limitation is that the study was conducted in a single center, which may raise concerns about the generalizability of the results. A further limitation is that the P2Y12 inhibitors prasugrel, ticagrelor, and clopidogrel were not investigated separately. Similarly, unfractionated heparin and low-molecular-weight heparin given for anticoagulation were not investigated separately. Subgroup studies could be designed to investigate the appropriateness of these agents separately. In centers without PCI, some drugs may not have been available for some periods and, therefore, appropriate treatment may not have been administered.

Since the pretreatment practitioners included many different types of clinicians (eg, general practitioners, family physicians, and emergency clinicians), no comparison was made. Finally, the low number of USAP diagnoses in the study may be due to the fact that the diagnosis was made solely on the basis of clinical anamnesis rather than definitive evidence, such as STEMI and NSTEMI. Neither did we investigate the impact of guideline compliance on clinical outcomes (eg, mortality, bleeding). Prospective studies with larger groups should be planned to address these limitations.

## CONCLUSION

The rates of compliance with both the previous and new European Society of Cardiology guidelines for pretreatment of acute cardiac syndrome by physicians working in hospitals without PCI centers were low. Pretreatment compliance during the new guideline period was lower than compliance during the previous guideline.

## Figures and Tables

**Table 1:** Patients’ demographic data and baseline characteristics of patients.

Characteristics	All Patients (N=1,450)
Sex	
Male, n (%)	1,013 (69.9)
Female, n (%)	437 (30.1)
Age (years), mean sd±	63.9 ± 13.00
Comorbidities	
Hypertension, n (%)	1,095 (75.5)
Diabetes, n (%)	494 (34.1)
Coronary artery disease, n (%)	559 (38.6)
Atrial fibrillation, n (%)	214 (14.8)
Chronic kidney disease, n (%)	194 (13.4)
Stroke, n (%)	119 (8.2)
Neoplasia, n (%)	92 (6.3)
Diagnostics	
USAP, n (%)	211 (14.6)
NSTEMI, n (%)	804 (55.4)
STEMI, n (%)	435 (30.0)
Pre-PCI Treatments	
Aspirin, n (%)	1,009 (69.6)
P2Y12 inhibitors, n (%)	930 (64.1)
Anticoagulation, n (%)	464 (32.0)
Mortality	
48 hours, n (%)	38 (2.6)
30 days, n (%)	69 (4.8)
Guideline Periods for Treated Patients	
Previous Guideline n (%)	684 (47.2)
New Guideline n (%)	766 (52.8)
Conformity to Guideline*, n (%)	142 (9.8)

* All pre-PCI treatments eligible.

*NSTEMI*, myocardial infarction without ST-segment elevation; *PCI*, percutaneous cardiac intervention; *STEMI*, myocardial infarction with ST-segment elevation; *PCI*, percutaneous cardiac intervention; *USAP*, unstable angina pectoris.

**Table 2:** Statistical analysis of the conformity of pre-percutaneous cardiac intervention treatments to the guideline between groups (previous and new European Society of Cardiology guidelines).

All Diagnostics	All Patients	Previous-Guideline Patients	New-Guideline Patients	P-Values
Conformity to aspirin, n (%)	1,009 (69.6)	453 (66.2)	556 (72.6)	.009
Conformity to P2Y12 inhibitors, n (%)	930 (64.1)	479 (70.0)	451 (58.9)	.001
Conformity to anticoagulation, n (%)	464 (32.0)	155 (22.7)	309 (40.3)	.001
Conformity to all Pre-PCI treatments, n (%)	142 (9.8)	79 (11.5)	63 (8.2)	.03
USAP				
Conformity to aspirin, n (%)	110 (52.1)	54 (50.0)	56 (54.4)	.52
Conformity to P2Y12 inhibitors, n (%)	181 (85.8)	92 (85.2)	89 (86.4)	.79
Conformity to anticoagulation, n (%)	16 (7.6)	8 (7.4)	8 (7.8)	.92
Conformity to all Pre-PCI treatments, n (%)	5 (2.4)	2 (1.9)	3 (2.9)	.67
NSTEMI				
Conformity to aspirin, n (%)	543 (67.5)	265 (66.9)	278 (68.1)	.71
Conformity to P2Y12 inhibitors, n (%)	570 (70.9)	288 (72.7)	282 (69.1)	.26
Conformity to anticoagulation, n (%)	221 (27.5)	85 (21.5)	136 (33.3)	.001
Conformity to all Pre-PCI treatments, n (%)	66 (8.2)	28 (7.1)	38 (9.3)	.24
STEMI				
Conformity to aspirin, n (%)	356 (81.8)	134 (74.4)	222 (87.1)	.001
Conformity to P2Y12 inhibitors, n (%)	179 (41.1)	99 (55.0)	80 (31.4)	.001
Conformity to anticoagulation, n (%)	227 (52.2)	62 (34.4)	165 (64.7)	.001
Conformity to all Pre-PCI treatments, n (%)	71 (16.3)	49 (27.2)	22 (8.6)	.001

*NSTEMI*, myocardial infarction without ST-segment elevation; *PCI*, percutaneous cardiac intervention; *STEMI*, myocardial infarction with ST-segment elevation; *USAP*, unstable angina pectoris.
